# The Role of Extracellular Vesicles in the Pathogenesis, Diagnosis, and Treatment of Osteoarthritis

**DOI:** 10.3390/molecules26164987

**Published:** 2021-08-17

**Authors:** Jianjing Lin, Li Wang, Jianhao Lin, Qiang Liu

**Affiliations:** 1 Arthritis Clinical and Research Center, Peking University People’s Hospital, No. 11 Xizhimen South Street, Beijing 100044, China; linjianjing@bjmu.edu.cn (J.L.); linjianhao@pkuph.edu.cn (J.L.); 2 Arthritis Institute, Peking University, Beijing 100044, China; 3 Department of Biomedical Engineering, College of Engineering, Peking University, Beijing 100871, China; wanglious@pku.edu.cn

**Keywords:** extracellular vesicles, osteoarthritis

## Abstract

Osteoarthritis (OA) is a degenerative joint disease that affects the entire joint and has been a tremendous burden on the health care system worldwide. Although cell therapy has made significant progress in the treatment of OA and cartilage regeneration, there are still a series of problems. Recently, more and more evidence shows that extracellular vesicles (EVs) play an important role in the progression and treatment of OA. Here, we discuss that EVs from different cell sources not only participate in OA progression, but can also be used as effective tools for the diagnosis and treatment of OA. In addition, cell pretreatment strategies and EV tissue engineering play an increasingly prominent role in the field of OA treatment. This article will systematically review the latest developments in these areas. As stated above, it may provide new insights for improving OA and cartilage regeneration.

## 1. Introduction

Osteoarthritis (OA) is a disease of the entire joint including structural changes in articular cartilage, subchondral bone, ligaments, joint capsule, synovium, and muscles around the joint [1,2]. OA affects 7% of the global population and more than 500 million people. From 1990 to 2019, the number of people globally affected increased by 48%. In 2019, osteoarthritis was the world’s 15th leading cause of disability, accounting for 2% of global causes of disability [3], which has been a tremendous burden on the health care system worldwide. Currently, efficient therapeutic strategies for OA treatment are still limited.

Cell therapy is considered to be a promising method for the treatment of OA and cartilage regeneration in which stem cells from different sources are widely used. However, there are still certain clinical impacts of the direct transplantation of stem cells into target tissues such as tumorigenicity, immune incompatibility, and chromosomal aberrations [4,5]. Because the regeneration, anti-inflammatory, immune regulation, and other functions of stem cells are mainly through their paracrine function [6], extracellular vesicles (EVs) have been widely studied. As intercellular communication carriers, EVs transfer biologically active lipids, proteins, and nucleic acids including mRNA, microRNA, and long-chain non-coding RNA (lncRNA) between cells, thereby triggering biological responses in recipient cells.

EVs cover various subtypes of membrane structures released by cells including exosomes, microvesicles, microparticles, tumor bodies, apoptotic bodies, and so on [7], which have different size and biogenesis. Although most of the current studies are conducted on exosomes, the physical characteristics of EVs overlap and are difficult to distinguish. Therefore, this review uses the collective term “EVs” to describe all of these vesicle populations.

In this review, we first introduce the role of EVs in the pathogenesis and diagnosis of OA. Then, we review the application of EVs from different cells and stem cell pretreatment strategies of EVs in the treatment of OA. Finally, we summarize the research progress of tissue engineering in EVs research related to OA.

## 2. The Role and Diagnostic Potential of EV in the Progression of OA

Chondrocytes, synovial cells, macrophages, subchondral bone, subpatellar fat pad, tendons, and ligaments can all secrete EVs in synovial joints [8]. Pathogenic signals are transmitted between different types of cells in the joints affected by OA through EVs, breaking the balance of the joint microenvironment and aggravating the development of OA. Samavedi et al. encapsulated normal/OA chondrocytes and activated macrophages in poly(ethylene glycol) diacrylate hydrogel for co-culture to simulate the natural 3D environment of the two types of cells in OA joints. The results indicated that chondrocytes expressed significantly higher levels of pro-inflammatory factors and matrix metalloproteinases (MMPs), indicating that activated macrophages may aggravate the abnormal matrix degradation and cytokine secretion associated with chondrocytes. Similarly, macrophages co-cultured with inflammatory chondrocytes express significantly more pro-inflammatory factors interleukin-1β (IL-1β) and vascular endothelial growth factor (VEGF), indicating that inflammatory chondrocytes may exacerbate abnormal macrophage activation [9]. Peng et al. found that THP-1 cell-derived M1 macrophages-EVs can induce the inflammatory response of temporomandibular joint (TMJ) chondrocytes by promoting the expression of inflammatory cytokines IL-8, IL6, and MMPs, and miR-1246-rich M1-EVs achieved this by inhibiting glycogen synthase kinase 3 beta (GSK3β) and axis inhibition protein 2 (Axin2) and activating the Wnt/β-catenin pathway [10]. Rossana Domenis et al. found that synovial fluid-derived exosomes from arthritis patients significantly stimulated M1 macrophages to release inflammatory cytokines IL-1β, chemokines chemokine (C-C motif) ligand 20 (CCL20), CCL15 and C-X-C motif chemokine ligand 1 (CXCL1), and MMP12 and MMP7, which can promote the inflammatory infiltration of chondrocytes and the destruction of cartilage extracellular matrix [11]. Gao et al. found that EVs from patients with end-stage knee OA had higher levels of cytokines, especially chemokines. Synovial fluid-derived EVs recruited inflammatory cells and inhibited cartilage proliferation, promoting joint degeneration [12]. EVs derived from vascular endothelial cells reduced the ability of chondrocytes to resist oxidative stress by inhibiting autophagy and p21 expression, thereby increasing cell ROS content and inducing cell apoptosis [13]. Wu et al. found that EVs derived from sclerotic osteoblasts inhibited the synthesis of the extracellular matrix (ECM) of chondrocytes and reduced the expression of chondrocyte-specific markers, and it is proved that miR-210-5p in EVs inhibited the oxygen consumption rate of chondrocytes and changed their bio-energy state, as often observed under OA conditions [14]. Ok Hee Jeon et al. found that senescent chondrocytes (SnCs) isolated from OA patients secreted more EVs than non-senescent chondrocytes, which inhibited the ECM deposition of healthy cartilage cells and can induce the senescence state of nearby cells. After removing SnCs, the composition of EV-related miRNA and proteins has changed significantly, reducing the development of OA and enhancing cartilage formation. This can be attributed to the differential expression of several specific miRNA (miR-30c, miR-92a, miR-34a, miR-24, miR-125a, miR-150, miR-186, and miR-223) and proteins (snake protein and polyprotein). Changes in the miRs and protein levels of synovial EVs are a potential mechanism for senescent cells to induce senescence of surrounding cells, inhibit tissue formation, and promote the development of OA [15]. Ni et al. found that OA chondrocyte-derived EVs can partially increase the production of mature IL-1β through miR-449a-5p/ATG4B-mediated autophagy inhibition, which may further aggravate synovitis and cartilage erosion in OA [16]. These data provide a new perspective for understanding the changes in the internal environment of OA.

The prevalence of OA in women is higher than that in men, and postmenopausal women have an increased risk of OA. Ravindra Kolhe et al. found that the female OA group had more miRNAs in knee synovial fluid than the male. Besides, miRNAs (miR-328-5p, miR-26a, hsa-miR-4654, miR-4707-5p, miR-4487, miR-24-3p, miR-6824-5p, miR-4740-5p, miR-8074, and miR-146a-5p) targeting toll-like receptor (TLR) and immune signaling pathways are downregulated in women with OA, while upregulated in men. It is speculated that it is precisely because of the increased expression of these miRNAs that the incidence and severity of OA in men are significantly lower than in women [17]. They also found that haptoglobin, oral mucin, and ceruloplasmin were significantly upregulated, while apolipoprotein was downregulated in female OA synovial fluid-derived EVs. Among them, haptoglobin and oral mucin will increase during inflammation and have been identified as an acute-phase glycoprotein [18]. Ceruloplasmin, as an antioxidant enzyme, is increased in the plasma of OA patients [19]. Apolipoprotein can effectively inhibit joint inflammation in rat OA model [20]. In conclusion, the changes in miRNA and protein content in synovial EVs of OA patients are gender-specific, which explains the increase in the prevalence and severity of OA in women.

OA is diagnosed through imaging and physical examination, which are relatively poor for the early diagnosis of OA. EVs reflect the physiological and pathological condition of cells, and may have the potential as diagnostic biomarkers for OA. At present, the conventional characterization methods of EVs mainly include western blot of specific markers, transmission electron microscopy (TEM), and nanoparticle tracking analysis (NTA). The same is true for OA-specific EV characterization techniques, as shown in Table 1. Since synovial fluid has a direct and close relationship with synovium, articular cartilage, and other knee joint tissues, synovial fluid can be used to monitor the pathophysiological changes of the joint space. Anne-Mari Mustonen et al. developed an applicable method based on confocal laser scanning microscopy (CLSM) and image analysis to quantify EVs, hyaluronic acid (HA)-particles, and hyaluronic acid-coated EVs (HA-EVs) in synovial fluid of human knee joints. The fluorescence intensity of EVs and HA-particles of RA was lower than that of the control group and the OA group, which were clearly separated by discriminant analysis based on CLSM data [21]. Zhao et al. found that the expression of EVs in the synovial fluid of OA patients was much higher than that of healthy people, but there was no significant difference between early OA and late OA. However, the expression of lncRNA PCGEM1 in EVs from synovial fluid is not only different between OA patients and healthy people, but also shows significant differences at different stages of OA. In addition, since the WOMAC index is highly correlated with the expression of lncRNA PCGEM1 in EVs, it is speculated that the severity of OA may be related to lncRNA PCGEM1, which may be a powerful indicator to distinguish early OA from late OA [22]. Studies have also found that compared with healthy people, LncRNA PVT1 in serum EVs of patients with osteoarthritis is significantly upregulated [23], while the level of miR-193b in plasma EVs of patients with OA is lower than that of healthy people [24].

## 3. Therapeutic Applications of EVs in OA

In recent years, more and more studies have shown that EVs from different types of cells have a definite therapeutic effect on OA and cartilage damage, as shown in Table 2.

### 3.1. Stem Cells

#### 3.1.1. Bone Marrow Mesenchymal Stem Cells (BMMSC)

A large number of studies have shown that BMMSC-EVs can inhibit the adverse effects of inflammatory mediators on cartilage homeostasis [25,26], which may be achieved by downregulating the pro-inflammatory Erk1/2, PI3K/Akt, p38, TAK1, and NF-κB signaling pathways [27,28]. Chen et al. found that the expression of E74-like factor 3 (ELF3) increased and miR-136-5p decreased in clinical samples of traumatic OA cartilage tissue. The BMMSC-EVs showed rich levels of miR-136-5p, and the expression of ELF3 in chondrocytes decreased after EV internalization. In a mouse model of post-traumatic OA, EV miR-136-5p was found to reduce the degradation of cartilage extracellular matrix. These data provided evidence that BMMSC-EVs miR-136-5p can promote chondrocyte migration in vitro and inhibit cartilage degeneration in vivo, thereby inhibiting OA [29]. Zhang et al. proved that BMMSC-EVs can promote the phenotypic transformation of synovial macrophages from M1 to M2, reduce synovial hyperplasia, and inflammatory cell infiltration, significantly reducing the levels of pro-inflammatory cytokines IL-1β and TNF-α [30]. Li et al. found that EVs derived from BMMSC can relieve pain by eliminating abnormal CGRP-positive nerves and abnormal H-type blood vessel formation in the subchondral bone of lumbar facet joint osteoarthritis in mice. Moreover, BMMSC-EVs alleviated cartilage degeneration and inhibited the expression of tartrate-resistant acid phosphatase and RANKL-RANK-TRAF6 signal activation to promote subchondral bone remodeling [31]. Zhou et al. obtained a special type of MSC from the bone marrow of the tissues removed from the hands of patients with polydactyly, and found that pBMSC-EVs stimulated the migration and proliferation of chondrocytes. Injection of pBMSC-EVs and BMSC-EVs reduced OA in the OA mouse model, but compared with BMSC-EVs, pBMSC-EVs had a better therapeutic effect. Therefore, pBMSC-EVs may represent a new OA treatment method [32].

#### 3.1.2. Adipose-Derived Mesenchymal Stem Cells (ADSC)

Since it is relatively feasible to obtain fat from patients through liposuction or arthroscopic surgery in clinic, EVs derived from adipose-derived mesenchymal stem cells may be a potential treatment for OA in the future. Wu et al. studied EVs derived from infrapatellar fat pad (IPFP) MSCs that improved the severity of OA, inhibited chondrocyte apoptosis, enhanced matrix synthesis, and reduced the expression of catabolic factors MMP13 and a disintegrin and metalloproteinase with thrombospondin motifs 5 (ADAMTS5). The mechanism may be related to the inhibition of the mTOR-autophagy pathway regulated by miR100-5p [33]. Carola Cavallo et al. emphasized the potential of ADSC-EVs to counteract IL-1β-induced inflammatory gene expression and protein release in chondrocytes and synovial cells [34]. Studies have found that intra-articular injection of ADSC-EVs significantly slowed down the progression of OA of mouse models and protects cartilage from degeneration. Besides, injection of hADSC-EVs inhibited the infiltration of M1 macrophages into the synovium [35]. Studies have also shown that EVs from ADSC downregulated the senescence-related β-galactosidase activity of OA osteoblasts and the accumulation of γ H2AX lesions. In addition, they reduced the production of inflammatory mediators IL-6 and prostaglandin E2 [36]. Zhao et al. found that co-culture of ADSC-EVs and activated synovial fibroblasts can downregulate the expression of pro-inflammatory markers IL-6, NF-κB, and tumor necrosis factor-α, and protect articular cartilage cells from apoptosis. EV treatment promoted the cartilage formation of periosteal cells and increased cartilage formation markers including type II collagen and β-catenin [37].

#### 3.1.3. Embryonic Mesenchymal Stem Cells (ESC)

Zhang et al. demonstrated that human ESC-EVs can promote the repair of critical-size osteochondral defects in an adult immunocompetent rat model for the first time [38]. Wang et al. found that intra-articular injection of ESC reduced cartilage destruction and matrix degradation in the destabilization of the medial meniscus (DMM) model through ESC-derived EVs [39]. Zhang et al. found that ESC-EVs enhanced the synthesis of sulfated glycosaminoglycan (s-GAG) blocked by IL-1β, and inhibited the production of nitric oxide and MMP13 induced by IL-1β. These effects are partially eliminated by adenosine receptor activation and inhibition of phosphorylation of AKT, ERK, and AMPK, which promotes the repair and regeneration of TMJ in OA [40]. Studies detected 200 secreted factors and 754 miRNAs in EVs of amniotic mesenchymal stem cells (AMSC). The analysis based on miRNAs related to OA and tendinopathy showed that the most abundant EV-miRNA can induce polarization of M2 macrophages, and then protect the tendon and cartilage. Overall, the presence of key regulatory molecules and miRNAs explains the promising therapeutic results of AMSCs and their secretion group in the treatment of musculoskeletal diseases [41].

#### 3.1.4. Other Stem Cells

Wang et al. found that the miR-31 packaged by synovial membrane MSCs-EV enhanced the proliferation and migration of chondrocytes, cartilage formation by targeting lysine-specific demethylase 2A (KDM2A), and improved knee OA [42]. Zhu et al. compared the effectiveness of EVs isolated from synovial membrane MSC (SMMSC-EVs) or induced pluripotent stem cell-derived MSCs (iMSC-EVs) for the treatment of OA, and found that iMSC-EVs had a better therapeutic effect than SMMSC-EVs in the collagenase-induced OA mouse model [43]. Luo et al. found that human deciduous tooth stem cell-EVs inhibited the expression of IL-6, IL-8, MMP1, MMP3, MMP9, and MMP13 as well as ADAMTS5 of TMJ chondrocyte through miR-100-5p/mTOR [44].

### 3.2. Other Cells

Wang et al. found that there were proteins in normal chondrocyte EVs that belonged to mitochondria and participated in immune system processes. Chondrocyte-derived EVs can restore mitochondrial dysfunction and polarize macrophages to the M2 phenotype. Intra-articular injection of EVs successfully prevented the development of OA [45]. Bai et al. co-cultured M2 macrophage EVs and chondrocytes to find that M2-EVs significantly increased cartilage-specific genes and proteins by delivering lncRNA MM2P to chondrocytes to promote cartilage repair [46].

### 3.3. Blood

Many basic, preclinical, and even clinical case studies and trials have reported the ability of platelet-rich plasma (PRP) to improve musculoskeletal diseases including osteoarthritis. However, due to the lack of standardization of PRP products, many people are not convinced by this result [47]. Isolated EVs from different cell sources have been suggested as therapeutic agents in OA cartilage recovery. Autologous EVs from blood products have advantages over cell-derived EVs because they do not require cell culture, do not involve cell therapy, do not cause uncontrolled differentiation of MSCs (such as undesired ossification) that may cause related adverse effects, and do not have risk of disease transmission from the donor to the recipient. Additionally, EV treatment can be standardized in terms of EV concentration compared with whole blood products [48]. Otahal et al. found that the EVs in citrate anticoagulated platelet-rich plasma (CPRP) and hyperacute serum (hypACT) were mainly derived from platelets, and the proportion of EVs derived from red blood cells and monocytes was very low. Compared with whole blood products, EV treatment of chondrocytes enhanced the expression of anabolic markers such as type II collagen, SRY-box transcription factor 9 (SOX9), and agglomerans, while preventing the release of pro-inflammatory cytokines. This highlighted the potential of autologous blood-derived EVs as a regulator of cartilage extracellular matrix metabolism and inflammation, and as a candidate for a new cell-free treatment of OA [48]. Liu et al. found that PRP-derived EVs (PRP-EVs) can significantly promote the proliferation and migration of chondrocytes and reduce the apoptotic rate of OA chondrocytes. PRP-EVs, as a carrier containing PRP-derived growth factors, provide a new treatment method for OA by activating the Wnt/β-catenin signaling pathway [49].

**Table 2:** Therapeutic applications of EVs in OA.

**Treated In Vitro**				
**Source**	**Cell(s) Used**	**Administration and Dose**	**Results**	**Reference**
BMMSC	Human chondrocytes	EVs were applied to cells at 600 μL.	BMMSC-derived EVs showed a rich level of miR-136-5p, which can be internalized by chondrocytes. MiR-136-5p promoted the migration of chondrocytes, increased the expression of collagen II, proteoglycan and SOX9, and decreased the expression of MMP-13. In addition, the expression of ELF3 in chondrocytes decreased after the internalization of EVs.	Chen et al., 2020 [29]
BMMSC	The murine macrophage RAW264.7 cell line treated with LPS (50 ng/mL) co-cultured macrophages with chondrocytes for 72 h	Cells treated with EVs for 24 h.	In EVs treatment group, the M2 ratio increased, and the levels of pro-inflammatory cytokines IL-1β, IL-6, and TNF-α were significantly reduced, while the levels of anti-inflammatory cytokine IL-10 were significantly increased. Besides, the expression of collagen X and runx2 decreased, while the expression of collagen II and sox9 increased.	Zhang et al., 2020 [30]
pBMMSC	Human chondrocytes	Cells treated with 1, 5, or 10 µg/mL EVs for 24 h.	EVs enhanced chondrocyte migration and proliferation	Zhou et al., 2020 [32]
ADSC	Human chondrocytes	Cells treated with 1, 5 and 10 × 10^8^ particles/mL EVs for 24 h.	EVs enhanced the expression of anabolic marker collagen II and decreased the expression of catabolic marker MMP13 and ADAMTS5. EVs can significantly increase the autophagy level of chondrocytes partially through mTOR inhibition.	Wu et al., 2020 [33]
ADSC	Human chondrocytes treated with IL-1β (10 ng/mL) for 18 h	Cells treated with 10 µg/mL EVs for 4 h and 15 h.	EVs significantly increased the expression of some cytokine/chemokine genes (IL-1β, MCP-1) and angiogenic factors (VEGF) in 4-h treatment group, but decreased the levels of IL-6, IL-8, MCP-1, MMP-1 in 15-h treatment group.	Cavallo et al., 2021 [34]
ADSC	Human chondrocytes treated with IL-1β (5 ng/mL) for 72 h	Cells treated with 1 × 10^8^ and 2 × 10^8^ particles/mL EVs for 72 h	EVs inhibited IL-1β-induced MMP production and significantly increased the mRNA expression of type II collagen inhibited by IL-1β in human OA chondrocytes.	Chang et al., 2020 [35]
ADSC	Human osteoblasts treated with IL-1β (10 ng/mL) for 24 h	Cells treated with 7.2 × 10^7^ particles/mL EVs for 7 days.	EVs downregulated the senescence-related β-galactosidase activity of OA osteoblasts and the accumulation of γ H2AX lesions that represent DNA damage. In addition, EVs reduced the production of inflammatory mediators IL-6 and prostaglandin E2.	Vian et al., 2017 [36]
ADSC	human chondrocytes treated with H_2_O_2_ (100 µM) for 1 h	Cells treated with 1, 5 and 10 × 10^10^ particles/mL EVs for 7 days.	ADSC-derived EVs inhibited apoptosis.	Zhao et al., 2017 [37]
ESC	Human chondrocytes treated with IL-1β (2 ng/mL) for 48 h	Cells treated with 1, 5 and 10 × 10^8^ particles/mL EVs for 24 h.	As the dose of EVs increased, Col II secreted by chondrocytes gradually increased, and ADAMTS5 decreased gradually.	Wang et al., 2017 [39]
ESC	Rat TMJ condylar chondrocytes treated with IL-1β (1 ng/mL) for 24 h.	Cells treated with 5 μg/mL EVs for 48 h.	EVs enhanced the synthesis of s-GAG blocked by IL-1β, and inhibited the production of NO and MMP13 induced by IL-1β.	Zhang et al., 2019 [40]
SMSC	Human chondrocytes	Cells treated with chondrocyte culture medium containing 600 μL EV for 12 h.	EVs can effectively promote the proliferation of chondrocytes. EVs loaded with miR-31 directly targeted KDM2A in chondrocytes to promote cartilage production.	Wang et al., 2020 [42]
iMSC	Human chondrocytes	Cells treated with 1 × 10^7^ and 1 × 10^8^ particles/mL EVs for 24 h and 48 h.	Both iMSC-EVs and SMMSC-EVs significantly enhanced the motility of chondrocytes, and iMSC-EVs was more effective than SMMSC-EVs in increasing motility for 24 and 48 h.	Zhu et al., 2017 [43]
Stem cells from human exfoliated deciduous teeth	human mandibular condyles chondrocytes treated with IL-1β (10 ng/mL) for 24 h	Cells pretreated with 150 μL (1.5 × 10^7^ particles) EVs for 2 h.	EVs inhibited the expression levels of pro-inflammatory cytokines IL-6 and IL-8 and proteases MMP1, MMP3, MMP9, MMP13, and ADAMTS5.	Luo et al., 2019 [44]
Chondrocyte	Mouse chondrocytes treated with IL-1β (10 ng/mL) for 72 h	Cells treated with EVs (200 μg/mL) for 48 h.	EVs reduced the protein levels of MMP13 and Adamts 5, and increased the expression levels of collagen II and proteoglycans, and eliminated mitochondrial dysfunction.	Zheng et al., 2019 [45]
Plasma- and serum-based autologous blood-derived products	Human chondrocytes treated with IL-1β (10 ng/mL)	Cells treated with medium supplemented with 1.42 × 10^9^ EVs.	EVs treatment of chondrocytes enhanced the expression of anabolic markers, such as type II collagen, SOX9 and aggregates, and prevented the release of pro-inflammatory cytokines NF-κB and COX2.	Otahal et al., 2020 [48]
Platelet-rich plasma (PRP)	Rabbit chondrocytes treated with IL-1β (10 ng/mL)	Cells treated with PRP-EVs (5 μg/mL or 50 μg/mL) for 24 h.	PRP-EVs promoted the proliferation and migration of chondrocytes through the Wnt/β-catenin signaling pathway, inhibited the release of TNF-α, and inhibited chondrocyte apoptosis.	Liu et al., 2019 [49]
**Treated In Vivo**				
**Source**	**Model(s) Used**	**Administration and Dose**	**Results**	**Reference**
BMMSC	A single mechanical load was applied to the mouse joint (1 mM/s to 12 N), causing the tibia to move forward relative to the femur and extended the anterior cruciate ligament beyond the point of failure.	Intra-articular injection of with 100 μL of 10^11^ particles/mL EVs immediately after the injury.	EVs miR-136-5p can reduce the degradation of cartilage extracellular matrix.	Chen et al., 2020 [29]
BMMSC	Rat model of OA: Modified Hulth technique: the anterior cruciate ligament was cut, and the medial meniscus was removed.	At 4 weeks post-operation, rats were injected intra-articularly with 10 ul of PBS or BMSC-derived EVs (10^10^ particles/mL) for 3 days for 4 weeks.	The cartilage degradation in the EVs group was less, the chondrocytes maintained close to normal morphology and distribution, and the number and surface area of osteophytes around the joints were smaller than those in the PBS group. The expression of hypertrophic genes, collagen X and runx2 was reduced. The expression of the cartilage forming genes type II collagen and sox9 increased. EVs inhibit cartilage degradation and osteophyte formation during the progression of rat OA.	Zhang et al., 2020 [30]
BMMSC	Mice were operated by resection of the lumbar 3rd–lumbar 5th (L3–L5) spinous processes along with the supraspinous and interspinous ligaments to induce instability in the lumbar spine.	The mice were injected weekly till four weeks right after surgery with EVs (200 μg of EVs in 200 μL PBS) via the tail vein.	EVs treatment significantly increased the expression of ACAN and decreased the expression of MMP13. BMSCs-EVs can attenuate abnormal nerve invasion and angiogenesis of subchondral bone, inhibit osteoclastogenesis by inhibiting the RANKL-RANK-TRAF6 signaling pathway in subchondral bone and promote cartilage and subchondral bone remodeling.	Li et al., 2020 [31]
pBMMSC	Collagenase-induced mouse model of OA.	The mice were injected with pBMSC-EVs on days 7, 14 and 21, respectively.	pBMSC-EVs can reduce cartilage damage caused by OA	Zhou et al., 2020 [32]
ADSC	Surgical destabilization of the medial meniscus (DMM).	The mice were given multiple intra-articular injections of 10 μL EVs (10^10^ particles/mL) for 4 weeks (twice a week).	EVs could enter the damaged area of articular cartilage, promote chondrocyte catabolism and inhibit its anabolism, and significantly prevented the cartilage destruction and partially improved the gait abnormality.	Wu et al., 2020 [33]
ADSC	Monosodium iodoacetate (MIA)-induced OA.	Starting from 5 weeks after DMM surgery, hASC-EVs (10^8^ particles in a volume of 6μL) were injected once a week for 6 weeks	EVs treatment effectively prevented proteoglycan degradation and prevented the progression of cartilage destruction after MIA injection.	Chang et al., 2020 [35]
ESC	Osteochondral defects were created on the trochlear grooves of both distal femurs in adult rats.	Intra-articular injection of exosomes (100 μg EVs per 100 μL injection) was performed after surgery, and subsequently on a weekly basis for 6 and 12 weeks.	Defects after EVs treatment showed complete recovery of cartilage and subchondral bone, with the characteristics including hyaline cartilage with good surface regularity, complete integration with adjacent cartilage, and extracellular matrix deposition that was very similar to the unoperated control.	Zhang et al., 2016 [38]
ESC	Surgical destabilization of the medial meniscus (DMM).	Inject 5 μL ESC-EVs (1 × 10^6^/joint) per joint, once every 3 days for 4 weeks.	Cartilage in the EVs group showed stronger Col II specific staining, much weaker ADAMTS5 specific staining, and proteoglycan specific staining than the PBS group.	Wang et al., 2017 [39]
ESC	MIA induced TMJ-OA.	Rats receive weekly intra-articular injections of 100 μg EVs in 50 μL PBS for 2, 4, or 8 weeks.	EVs reduced the expression of IL-β and iNOS during the repair of TMJ, and reduced the degeneration of subchondral bone in TMJ-OA.	Zhang et al., 2019 [40]
SMSC	OA model was established by completely transecting the medial collateral ligament and medial meniscus, cutting the meniscus at the narrowest point without damaging the tibial surface, and transecting the anterior cruciate ligament.	5 μL SMSC-EV particles per mL were injected into the articular cavity on the first day of each week from the 5th to the 8th week after surgery.	EVs encapsulated miR-31 inhibited knee joint OA through the KDM2A/E2F1/PTTG1 axis.	Wang et al., 2020 [42]
iMSC	Collagenase-induced mouse model of OA.	The mice were injected intra-articularly with 8 μL iMSC-EVs (10^10^/mL) on days 7, 14 and 21.	EVs group had stronger type II collagen expression and safranine staining than SMMSC-EVs group.	Zhu et al., 2017 [43]
Chondrocyte	Anterior cruciate ligament transection (ACLT).	10 days after surgery, mice received intra-articular injections of 200 μg exosomes every week	Almost complete preservation of both femoral and tibial cartilage and effective prevention of the development of OA were showed in EVs group. EVs can increase the infiltration of M2 macrophages in the synovium, while the M1 macrophages decrease.	Zheng et al., 2019 [45]
M2 macrophage	Surgical destabilization of the medial meniscus (DMM).	One week after DMM surgery, mice were treated by tail vein injection.	EVs injection reversed abnormal subchondral bone formation and sclerosis.	Bai et al., 2020 [46]
Platelet-rich plasma (PRP)	Resection of the medial collateral ligament, anterior cruciate ligament (ACL) and medial meniscus of the left knee.	Inject 100 μg/mL PRP-EVs into the joint cavity once a week for 6 weeks.	PRP-EVs reversed the decrease in collagen II and RUNX2 protein expression caused by OA, promoted cartilage repair, and inhibits osteoarthritis	Liu et al., 2019 [49]

## 4. Cell Pretreatment Strategy

Studies have shown that pretreatment is an effective way to improve the ability of cells to resist adverse microenvironments. Stem cell pretreatment can improve cell survival and differentiation potential, regulate immune response, inhibit fibrosis, and enhance cell secretion of anti-inflammatory factors [50]. Therefore, a stem cell pretreatment strategy has also received a lot of attention in the field of releasing EVs to treat OA.

### 4.1. Physical Factors

The strategy of using physical factors to stimulate cells to produce EVs has been tried in the OA and cartilage fields. Research has shown that LIPUS can promote BMSC cartilage formation [51,52]. Liao et al. studied the effect of LIPUS combined with BMSC-EVs on OA cartilage and concluded that LIPUS promoted BMSC-EVs to inhibit OA inflammation, and further promoted its increase in cartilage extracellular matrix synthesis and chondrocyte proliferation, promoting the regeneration of articular cartilage [53]. Although physical stimulation of cells to produce EVs is rarely used in the treatment of OA/cartilage injury, we still believe that this strategy is of great value.

### 4.2. Pharmacological Agents

Due to the low solubility, instability, and rapid systemic elimination, the bioavailability of a large number of drugs is low. Therefore, the use of drugs to pretreat stem cells and improve the therapeutic effect of EVs on OA is another pretreatment strategy. Research by Li et al. showed that compared with the control BMSC and free curcumin, EVs derived from curcumin treated BMSC (Cur-EVs) can significantly reduce the catabolic genes in OA chondrocytes induced by IL-1β, and increase the expression of anabolic genes. These effects may be partly attributed to the upregulated expression of hsa-miR-126-3p in target cells induced by Cur-EVs, which results in reduced phosphorylation of pro-inflammatory signaling pathway components [54]. Qiu et al. found that miR-143 and miR-124 were upregulated in the mouse OA model after treatment of EVs derived from curcumin-treated MSC, and the normal expression of NF-kB and Rho-associated coiled-coil containing protein kinase 1 (ROCK1) was significantly restored, resulting in weakened progression of OA [55]. Studies have shown that EVs isolated from rabbit subpatellar fat pad mesenchymal stem cells pretreated with Kartogenin (KGN) have the effective ability to induce chondrogenic differentiation of stem cells, effectively promote the proliferation and expression of chondrogenic proteins and genes of chondrocytes, and are more effective to promote the repair of articular cartilage defects [56]. Similarly, EVs derived from KGN pretreated SD rat BMSC are more effective than EVs derived from BMSC in cartilage repair [57]. Jing et al. evaluated the regenerative potential of sEVs from human umbilical cord mesenchymal stem cells (hUCMSCs) pretreated with KGN to induce cartilage differentiation of MSCs, which was mainly achieved by providing sEV-miR-381-3p targeting thousand and one kinase 1 (TAOK1) [58].

### 4.3. Biological Factors

Wang et al. found that TGF-β1 stimulation can upregulate the expression of miR-135b in MSC-EVs, and delivering TGF-β1 pretreated MSC-EVs to chondrocytes can downregulate Sp1 to promote chondrocyte proliferation, thereby promoting cartilage repair in OA [59]. They also found that MSC-EVs pretreated with TGF-β1 can reduce the upregulation of pro-inflammatory factors in the serum of OA rats and the damage of cartilage tissue through miR-135b that target MAPK6 to promote the M2 polarization of synovial macrophages [59]. There are also studies on directly modifying EVs with biological factors for OA treatment. Activated transcription factor 4 (ATF4) is essential for chondrocyte proliferation and bone formation. Wang et al. introduced ATF4 mRNA into serum-derived EVs from OA mice (OA-EVs) by electroporation, and found ATF4-modified OA-EVs can protect cartilage and alleviate OA progression by inducing autophagy [60].

### 4.4. Genetic Modification

It is well known that non-coding RNA (microRNA [miRNA] and long-chain non-coding RNA [lncRNA]) is currently one of the most studied molecules in EV research. Qiu et al. found that MSC-EVs overexpressing miR-129-5p inhibited IL-1β-mediated upregulation of HMGB1 and significantly reduced the inflammation and apoptosis of chondrocytes [61]. EVs of synovial mesenchymal stem cells (SMSCs) overexpressing miR-140-5p (SMSC-140-EVs) enhanced the proliferation and migration of chondrocytes in vitro without destroying the secretion of ECM, while in vivo, SMSC-140-EVs successfully prevented OA in the rat model [62]. EVs from human MSCs overexpressing miR-92a-3p enhanced cartilage formation and inhibited cartilage degradation by targeting WNT5A [63]. EVs from SMSCs overexpressing miR-155-5p prevented osteoarthritis by enhancing proliferation and migration, reducing cell apoptosis, and regulating extracellular matrix secretion in chondrocytes [64]. EVs from synovial fibroblasts overexpressing miR-126-3p inhibited chondrocyte inflammation and cartilage degradation in a rat model of OA [65]. MiR-26a-5p-overexpressed HBMSC-derived EVs delayed the damage of synovial fibroblasts in vitro and reduced OA damage in vivo [66]. Mao et al. found that the expression of miR-95-5p in EVs secreted by OA chondrocytes was significantly lower than that of normal cartilage and EVs derived from primary chondrocytes overexpressing miR-95-5p (AC-miR-95-5p-EVs) could enhance cartilage formation and prevent the development of OA by directly targeting HDAC2/8 [67].

Liu et al. found that transplantation of EVs secreted by stem cells overexpressing the novel lncRNA KLF3 Antisense RNA 1 (lncRNA KLF3-AS1) could promote cartilage repair by promoting chondrocyte proliferation and inhibiting cell apoptosis through the miR-206/GIT1 axis, emphasizing the possible mechanism of OA treatment through cellular delivery of lncRNA KLF3-AS1 [68]. Tan et al. found that fibroblast-like synoviocytes overexpressing LncRNA H19 (FLS-LncRNA H19) had a positive effect on reversing IL-1β-induced chondrocyte damage. FLS-LncRNA H19-derived EVs can promote cell viability and migration, and prevent IL-1-β-induced ECM degradation in chondrocytes by regulating the expression of miR-106b-5p and Tissue inhibitor of metalloproteinases 2 (TIMP2) [69].

### 4.5. Hypoxia

The oxygen content of the physiological environment in vivo is very different from the oxygen content observed in the growth medium (21% O_2_ in normoxia). Therefore, the experimental parameters in vitro may not be able to simulate the real microenvironment in vivo. However, the function of hypoxia in EVs is controversial with evidence showing the adverse effects of EVs after hypoxia. Ding et al. found that EVs secreted during hypoxia in synovial fibroblasts (SFs) aggravated rheumatoid arthritis by regulating Treg/Th17 balance [70]. However, research by Hu et al. showed that when cells were cultured at an oxygen concentration lower than 0.5%, hypoxic pretreatment (HP) increased MSC migration and reduced MSC apoptosis [71]. In terms of OA and cartilage repair, in rabbit knee joint trauma and focal early OA models, aerobic MSC treatment significantly improved cartilage repair scores compared with hyperoxic MSC and their respective control defects [72]. Rong et al. found that EVs derived from BMSCs treated with hypoxia-inducible factor 1α-induced hypoxic can promote chondrocyte proliferation, migration, and inhibit apoptosis through the miR-216a-5p/JAK2/STAT3 signaling pathway as well as mediate cartilage repair in osteoarthritis [73].

## 5. EV Delivery Biomaterials

The progress of OA has complicated the delivery of EVs in the joint cavity. However, regardless of the inflammation state of the joint, 300 nm nanoparticles can quickly escape from the joint space [74]. Unfortunately, some of the EVs (such as exosomes) are less than 300 nm, which are difficult to keep in the joint space for a long time. Therefore, it is crucial to develop an intra-articular injection device for the controlled release of EVs.

Yang et al. developed a Diels–Alder crosslinked hyaluronic acid (HA)/polyethylene glycol (PEG) hydrogel (DAHP) for intraarticular MSC-EV delivery. Through the controlled release, the effect of EVs in improving OA was enhanced, and the average OARSI score of the EV-loaded DAHP hydrogel-treated group was low, which showed no significant difference to that of the multiple EV injection group, but was lower than that of the single EV injection group, the DAHP hydrogel group, and the saline group [75]. Liu et al. developed a photoimine cross-linked hydrogel as an EV scaffold for cartilage regeneration, while most of the encapsulated EVs can still be retained in the hydrogel (>90%) after being immersed in PBS for 14 days. The controlled release system had active cell regulation in vitro and in vivo, and promoted cartilage repair and regeneration [76]. Chen et al. used desktop stereolithography to fabricate a 3D printed cartilage extracellular matrix (ECM)/gelatin methacrylate (GelMA)/EV scaffold with radially oriented channels. The scaffold can continuously release EVs for at least 14 days in vitro, and retain EVs in vivo for at least seven days [77]. Hu et al. prepared a GelMA/nanoclay/EV hydrogel. EVs were released in a sustained manner as the hydrogel degraded, achieving successful cartilage regeneration, exhibiting intact and smooth regenerated neotissue, which were well integrated with the surrounding original cartilage and similar to the normal cartilage [78]. Tao et al. used the thermosensitive hydrogel PDLLA-PEG-PDLLA triblock copolymer gel to show good performance in sustained-release EVs. Compared with the direct injection of EVs, it had better long-term effects on the protection of cartilage and delaying the progression of OA [79].

## 6. Engineered EV Strategy

The use of engineered strategies to improve EVs has been extensively studied in the field of oncology (such as prostate cancer [80], breast cancer [81]) and rheumatoid arthritis [82]. The strategy has gradually received attention in the treatment of OA/cartilage injury.

The densely packed collagen and proteoglycan and highly negatively charged glycosaminoglycan in the joint tissue form a barrier for the drug to reach the chondrocytes, which makes cartilage cell targeted drug delivery a daunting task. Liang et al. fused the lysosomal-associated membrane protein 2 (LAMP-2B) gene with chondrocyte affinity peptide (CAP) and transfected it into dendritic cells to generate EVs that target chondrocytes, and miR-140 was delivered to chondrocytes in the deep area of cartilage to extend retention in the cartilage area and induce cartilage repair [83]. Xu et al. fused the MSC-binding peptide E7 with the EV membrane protein Lamp 2b to produce EVs with E7 peptides (E7-EVs) with synovial fluid mesenchymal stem cell (SF-MSC) targeting ability. Compared with KGN delivered alone or by EVs without E7, KGN delivered by E7-EVs effectively enters SF-MSC and induces a higher degree of cartilage differentiation, which is expected to become an advanced stem cell therapy for OA [84].

## 7. Conclusions

**Figure 1:**
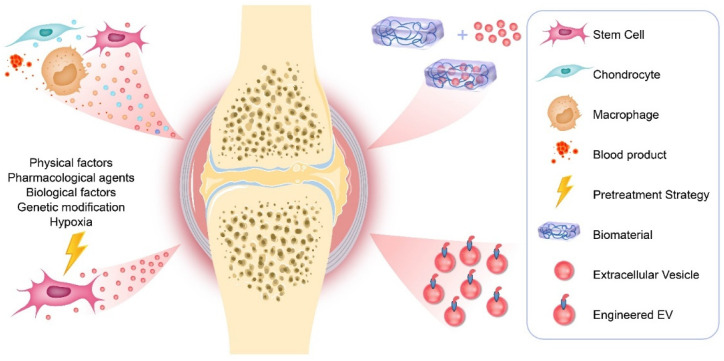
Therapeutic applications of EVs in OA.

EVs are natural nanoparticles containing proteins, mRNA, miRNA, LncRNA, and lipids, which participate in the communication between cells. In recent years, they have received more and more attention in the OA field. EVs from different cell sources not only participate in the progress of OA, but can also be used as an effective tool for the diagnosis and treatment of OA. In addition, the development of cell pretreatment strategies and tissue engineering technology has achieved encouraging results as summarized in Figure 1, where EVs may open up new strategies for the treatment of OA.

## Figures and Tables

**Table 1:** Characterization methods of OA-specific EVs.

Source	NTA	TEM	Western Blot	Flow Cytometry	Reference
Synovial fluid	The majority of the particles were within the range size of 100 nm.		CD9, CD63, CD81, and TSG101	CD9, CD81, and CD63	Domenis et al., 2017 [11]
Synovial fluid	The particles were with the range size of 100–125 nm. KL 3–4 SF contained more exosome particles than that of KL 1–2.	Round vesicles with an approximate diameter of 50–200 nm	CD9, CD63, and CD81		Gao et al., 2020 [12]
Synovial fluid	EV size and concentration in synovial fluid from both OA and normal donors were similar				Jeon et al., 2019 [15]
Synovial fluid	The size and concentration of EVs are almost similar between OA and NON-OA patient’s synovial fluid.	~100 (±10) nm diameter size ranges.	Tsg101, CD63, and CD81		Kolhe et al., 2021 [17]
Synovial fluid and plasma	EVs isolated from plasma and synovial sample were in size with 100 nm	Round-shaped or oval-shaped in morphology.	CD9, CD63, and TSG101	The expression of exosomal lncRNA PCGEM1 from synovial fluid in early OA and late-stage OA was markedly higher than that in controls.	Zhao et al., 2018 [22]
Serum	The exosomes were circular in shape and 40–100 nm in the C28/I2 cells and the serum of OA patients and healthy volunteers		CD9 and CD63		Meng et al., 2020 [23]
Plasma	Most of these vesicles ranged from 50 to 200 nm in size	Hollow spherical microvesicles.	CD63 and CD9		Meng et al., 2018 [24]

## Data Availability

Not applicable.
